# Structure-Activity Relationships for the Antifungal Activity of Selective Estrogen Receptor Antagonists Related to Tamoxifen

**DOI:** 10.1371/journal.pone.0125927

**Published:** 2015-05-27

**Authors:** Arielle Butts, Jennifer A. Martin, Louis DiDone, Erin K. Bradley, Mitchell Mutz, Damian J. Krysan

**Affiliations:** 1 Department of Chemistry, University of Rochester, Rochester, NY 14642, United States of America; 2 Department of Pediatrics, University of Rochester, Rochester, NY 14642, United States of America; 3 Department of Microbiology/Immunology, University of Rochester, Rochester, NY 14642, United States of America; 4 LigDCipher, San Diego, CA 92121, United States of America; 5 Amplyx Pharmaceutical, San Diego, CA 92121, United States of America; Yonsei University, REPUBLIC OF KOREA

## Abstract

Cryptococcosis is one of the most important invasive fungal infections and is a significant contributor to the mortality associated with HIV/AIDS. As part of our program to repurpose molecules related to the selective estrogen receptor modulator (SERM) tamoxifen as anti-cryptococcal agents, we have explored the structure-activity relationships of a set of structurally diverse SERMs and tamoxifen derivatives. Our data provide the first insights into the structural requirements for the antifungal activity of this scaffold. Three key molecular characteristics affecting anti-cryptococcal activity emerged from our studies: 1) the presence of an alkylamino group tethered to one of the aromatic rings of the triphenylethylene core; 2) an appropriately sized aliphatic substituent at the 2 position of the ethylene moiety; and 3) electronegative substituents on the aromatic rings modestly improved activity. Using a cell-based assay of calmodulin antagonism, we found that the anti-cryptococcal activity of the scaffold correlates with calmodulin inhibition. Finally, we developed a homology model of *C*. *neoformans* calmodulin and used it to rationalize the structural basis for the activity of these molecules. Taken together, these data and models provide a basis for the further optimization of this promising anti-cryptococcal scaffold.

## Introduction

Invasive fungal infections pose a significant and increasingly prevalent, global health care challenge. In part, this is due to the growing number of people who are living with compromised immune function and are, consequently, susceptible to infections from opportunistic pathogens such as fungi [1]. Of the invasive fungal infections, cryptococcosis is one of the most significant causes of human fungal disease world-wide [2]. Human cryptococcosis is caused *C*. *neoformans* var. *grubii*, *C*. *neoformans* var. *neoformans* and *C*. *gattii* with *C*. *neoformans* var. *grubii* causing the majority of disease. *Cryptococcus neoformans* causes an estimated 1 million new invasive infections every year resulting in approximately 650,000 deaths [2]. The vast majority of cryptococcosis occurs in patients living with HIV/AIDS and, as such, it is one of the most common causes of infectious disease-related death in this patient population. While cryptococcosis occurs in immuno-compromised individuals primarily, it is important to note that the ongoing outbreak of cryptococcosis in Vancouver and the western United States caused by *C*. *gattii* [3] has affected individuals with no identifiable immune deficiency.

Cryptococcosis manifests primarily as meningoencephalitis and is invariably fatal if not treated [4]. The gold standard therapy for cryptococcal meningoencephalitis is amphotericin B (AMB) combined with flucytosine (FC) and, as demonstrated in a recent clinical trial, is more effective than AMB alone [5]. AMB/FC is fungicidal and leads to clearance of the pathogen from the CSF. The drawbacks to this therapy are: 1) intravenous medication-based requiring hospitalization (AMB); 2) toxicities requiring laboratory monitoring (AMB/FC); and 3) poor availability of the drug in resource-limited regions (FC). As a result, AMB/FC is not widely available in resource-limited regions of the world without strong medical infrastructures [6]. In many of these regions, the alternative therapy is fluconazole which is available by donation from its manufacturer, is orally administered, and very well-tolerated. Fluconazole, however, is much less effective than AMB/FC. The decreased efficacy of fluconazole is due in large part to the fact that it is a fungistatic drug and, consequently, does not rapidly clear Cryptococcus from the central nervous system [7]. The ability of a drug to clear Cryptococcus from the cerebrospinal fluid is referred to as early fungicidal activity (EFA) and correlates with patient outcome [7]. AMB/FC has the highest EFA of therapies currently in clinical use. As noted above, AMB/FC is not available in many regions of the world with high burdens of cryptoccocal disease. Fluconazole, on the other hand, is widely available, safe, and easily administered because of its oral bioavailablility. It is likely that reliance on this less efficacious agent is partly responsible for the higher mortality associated with cryptococcosis in resource-limited regions [6]. Consequently, new therapies for cryptococcosis that are fungicidal and that can be widely applied are needed [8, 9].

The pressing need for new antifungals has dovetailed with a growing focus on drug repurposing [10]. The goal of repurposing is to expedite the drug development process by identifying new biological activities for existing drugs and then applying those drugs to the treatment of a new disease. The advantage of repurposing is that the drug or scaffold has known pharmacological and toxicological properties in humans. Consequently, the timeline for translation from bench-to-beside for such drugs can be compressed. Although the ideal result of a repurposing approach is to identify an approved drug that can be directly used for a new indication without changes in dosing or formulation, the drug can also be useful as a lead compound upon which to design derivatives optimized for the newly identified activity. This is particularly attractive if the drug has pharmacological and toxicological properties that are advantageous for the treatment of the new condition. Indeed, it can be easier to optimize the biological activity of a drug for a new target than to improve the toxicology or pharmacologic properties of a given molecular scaffold.

**Fig 1:**
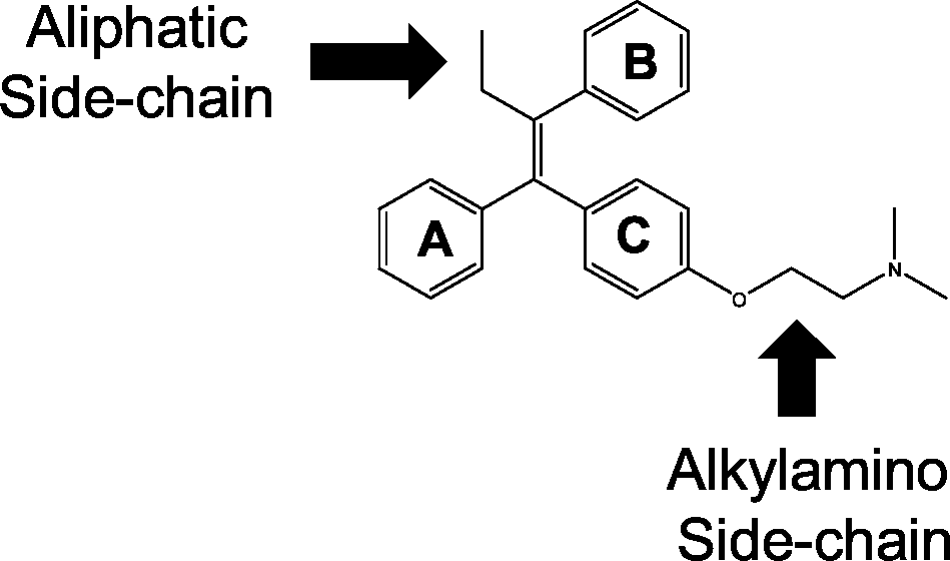
Structure of the tamoxifen-derived triphenylethylene scaffold with key regions of the molecule annotated.

As part of a recent repurposing effort, our laboratory screened a collection of FDA-approved compounds for fungicidal activity against *Cryptococcus neoformans* [11]. Of the hits from this screen, triphenylethylene-based selective estrogen-receptor modulators (SERM) related to tamoxifen (Fig 1) emerged as attractive anti-cryptococcal candidates [12]. In addition to being fungicidal against *C*. *neoformans*, tamoxifen and related molecules have three properties that are advantageous for the treatment of cryptococcal meningitis. First, these drugs have excellent bioavailability and, therefore, can be administered orally [13]. Second, the triphenylethylene class of drugs penetrates the blood-brain barrier [14], an absolute requirement for any anti-cryptococcal drug candidate. Furthermore, the lipophilic nature of this class of compounds facilitates accumulation in tissues and tamoxifen concentrates in the brain to levels 20–40-fold higher than the levels in the serum (14). Third, we have shown that tamoxifen and toremifene are active against *C*. *neoformans* within macrophages [12]. Macrophages appear to be an important niche for *C*. *neoformans* and their ability to replicate with the phagolysosome may contribute to dissemination from the lung to the brain [15]. Neither AMB/FC nor fluconazole are active against intra-phagocytic *C*. *neoformans* [16]. Based on these properties, we were interested in examining the structure-activity relationships of the tamoxifen scaffold with respect to its antifungal properties.

The antifungal activity of tamoxifen was first identified 25 years ago by Wiseman et al. [17]. We and others have further characterized its activity and mechanism of action since that time [18, 19, 20]. Importantly, we have shown that tamoxifen has activity in mouse models of disseminated cryptococcosis and candidiasis. We have also shown that the antifungal activity of tamoxifen is due, at least in part, to its ability to inhibit calmodulin [12, 18]. However, despite the relatively large number of estrogen receptor antagonists that are commercially available, the generality of the antifungal activity of this class of drugs has not been extensively explored beyond the small set of molecules described in Butts et al [12]. Here, we report the anti-cryptococcal activity of: a) a collection of commercially available, structurally diverse SERMs and b) a series of tamoxifen derivatives obtained from the archive of AstraZeneca, the maker of tamoxifen. From these data, we have identified structural features of the tamoxifen scaffold that contribute to its anti-cryptococcal activity and provide additional data indicating that antifungal activity of this scaffold correlates with calmodulin inhibition. Finally, we have developed a homology model of *C*. *neoformans* calmodulin based on the structure of the bovine calmodulin-tifluoperazine complex as an approach to understanding the protein-ligand interactions contributing to the structure-antifungal activity relationships of the tamoxifen and its derivatives.

## Results

### The anti-cryptococcal activity of the triphenylethylene scaffold is general

**Fig 2:**
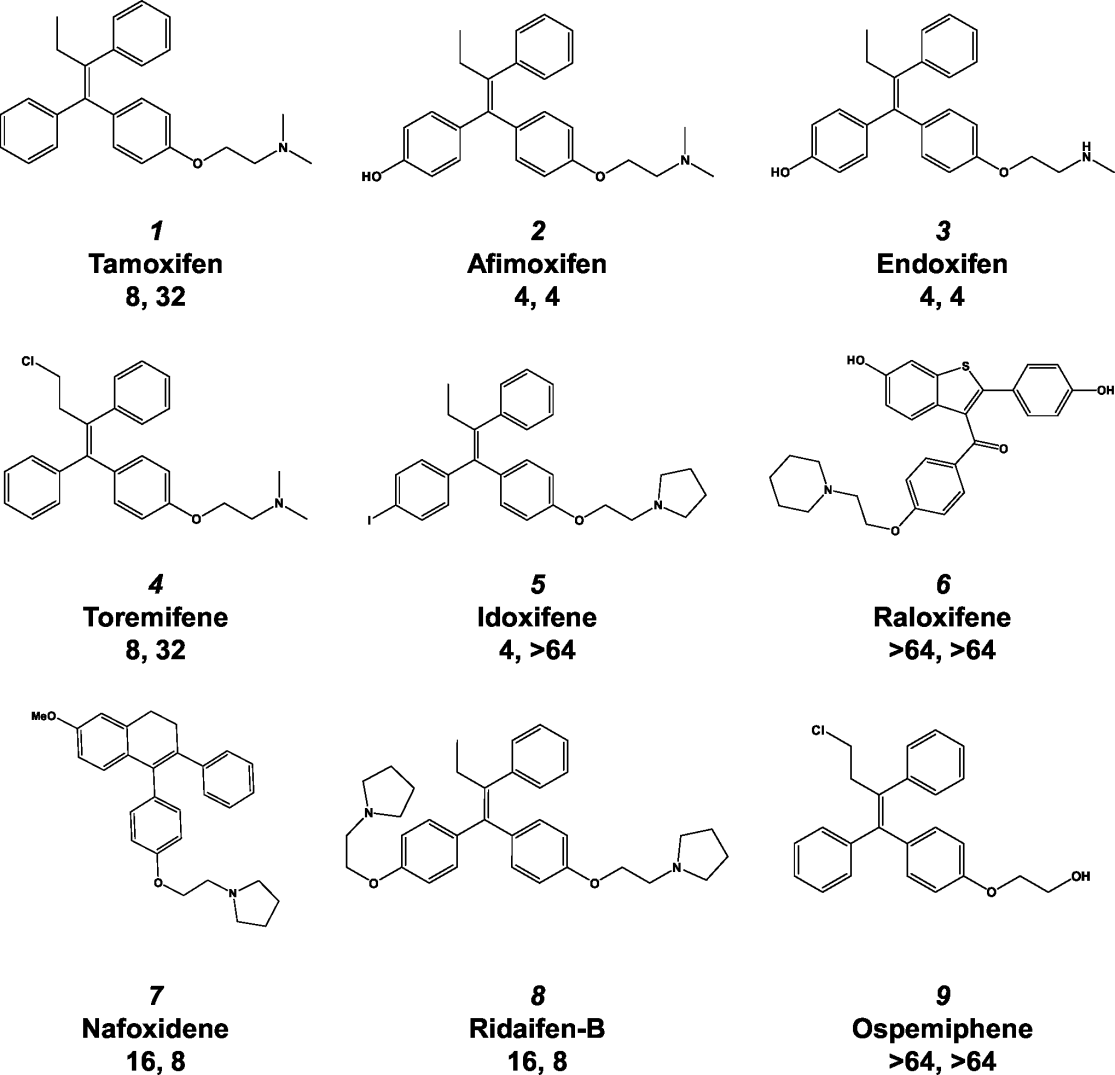
Activity of commercially available SERM against *C*. *neoformans* and *C*. *albicans*. The structure, molecule number used in the text, name, minimum inhibitory concentration (MIC, μg/mL) against *C*.*neoformans* (first number) and MIC against *C*. *albicans* are provided.

We assembled a collection of commercially available, structurally-distinct estrogen receptor antagonists (Fig 1) and determined their activity against *C*. *neoformans* and *C*. *albicans* using standardized CLSI methods. We included previously reported [12, 18] data for tamoxifen, its metabolites endoxifen and hydroxy-tamoxifen, toremifene and idoxifen (Fig 2, molecules 1–5). Tamoxifen and toremifene are less active against *C*. *albicans* as compared to *C*. *neoformans*. We have also previously shown that the two major metabolites of tamoxifen, 4-hydroxy-tamoxifen (Afimoxifen, Fig 2, molecule 2) and endoxifen (Fig 2, molecule 3) are more active against *C*. *neoformans* than the parent drug [12]. Against *C*. *albicans*, the improved activity of the metabolites relative to the parent tamoxifen is more dramatic; the MIC for afimoxifen (molecule 2) and endoxifen (molecule 3) is 8-fold lower than tamoxifen. The improved activity of the afimoxifen and endoxifen metabolites may explain why tamoxifen is efficacious in a mouse model of disseminated candidiasis despite its relatively poor MIC [18]. The molecules with improved activity against *C*. *albicans* relative to tamoxifen (Fig 2, molecules 2, 3, 7, and 8) have hydroxyl- or alkoxyl- substituents at the 4-position of ring A (Fig 1), suggesting that this moiety is particularly important for anti-candidal activity.

A second general class of SERMs is derived from the tamoxifen scaffold and incorporates the central ethylene portion of the molecule into a ring system. To examine the effect of these ring systems, we tested the antifungal activity of cyclic SERMs. First, we examined raloxifene which has the ethylene group embedded in a thiophene moiety. As shown in Fig 2 (molecule 6), raloxifene has no activity against either *C*. *neoformans* or *C*. *albicans*. Since raloxifene is a potent SERM, this observation indicates that the structural features of the scaffold that determine its antifungal activity are not the same as those that effect estrogen receptor binding. Nafoxidine is a SERM in which the double bond is incorporated into a dihydronapthalene ring system (Fig 2, molecule 7). The activity of nafoxidine is identical to tamoxifen with respect to *C*. *neoformans* and 2-fold more active than tamoxifen against *C*. *albicans*, indicating that the six-membered ring system does not interfere with antifungal activity.

An additional feature of the SERM scaffold is the aliphatic linker that tethers a basic amine moiety to ring C (Fig 1). All but one of the analogs that we tested has a two carbon chain that appends the basic amine to ring C through a phenoxy group. Derivative with dimethylamino-, methylamino-, and pyrrolidino- groups are all active (Fig 2: molecules 3, 4, 5). Replacement of the amine with a hydroxyl group eliminates the antifungal activity of the molecule as shown by the fact that the ospemiphene, a hydroxyl containing derivative of toremifene, has an MIC > 64 μg/mL against both *C*. *neoformans* and *C*. *albicans* (Fig 2, molecule 9). Placement of alkoxyamino groups on rings A and C (Ridaifen-B, Fig 2, molecule 8) reduces the anti-cryptococcal activity slightly but improves anti-candidal activity four-fold. Thus, the presence of an alkoxyalkyl amino group is crucial for the antifungal activity of the tamoxifen scaffold. Once again, this functionality is not required for SERM activity and further supports the conclusion that the structural determinants of the scaffold’s antifungal activity are different from those that determine its estrogen receptor binding activity.

### The anti-cryptococcal activity of tamoxifen is improved by increasing the electronegative properties of the aromatic ring systems

**Fig 3:**
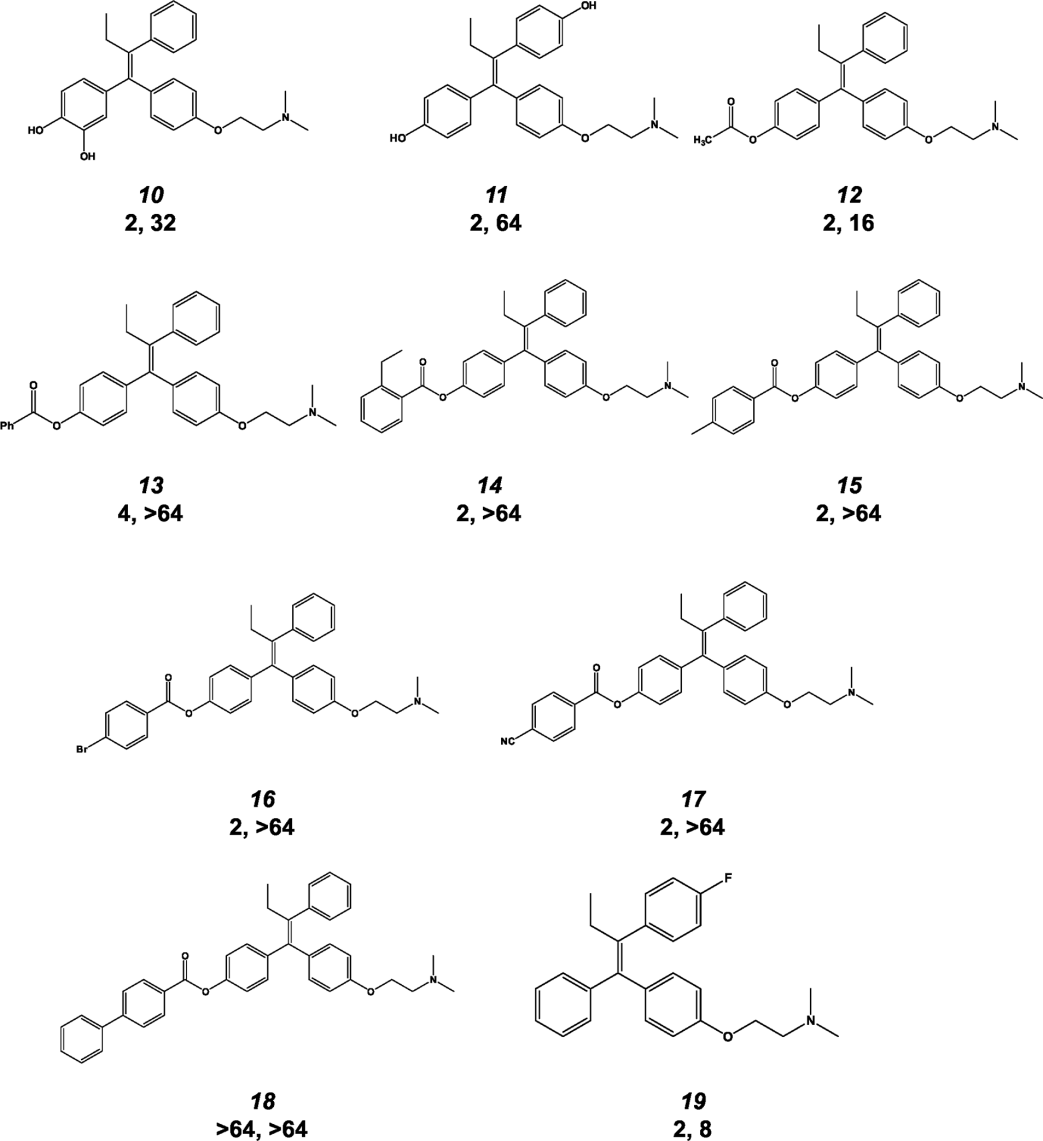
Antifungal activity of aryl-ring substituted tamoxifen derivatives. The structure, molecule number used in the text, minimum inhibitory concentration (MIC, μg/mL) against *C*.*neoformans* (first number) and MIC against *C*. *albicans* are provided.

To further characterize the antifungal activity of triphenylethylene-SERMs, we obtained a collection of thirteen tamoxifen analogs from the chemical archives of AstraZeneca. The majority of these molecules are derivatives of 4-hydroxy-tamoxifen; the activity of this series of analogs is summarized in Fig 3. Relative to 4-hydroxy-tamoxifen (afimoxifene, Fig 2, molecule 2, MIC 4 μg/mL), introduction of a second hydroxyl group at the 3-position of 4-hydoxy-tamoxifen ring leads to a 2-fold increase in activity against *C*. *neoformans* (Fig 3, molecule 10) but decreases activity against *C*. *albicans* (MIC 32 μg/mL). The afimoxifeme derivative containing a hydroxyl-group at the 4-position of ring B was also 2-fold more active against *C*. *neoformans* (Fig 3, molecule 11) than afimoxifene (molecule 2). Thus, hydroxyl substituents in either ring A or B improves the activity of the tamoxifen scaffold against *C*. *neoformans*.

The collection of tamoxifen analogs contained seven ester derivatives of 4-hydroxy-tamoxifen/afimoxifene (molecule 2). Five of the seven ester derivatives were 2-fold more active than the parent 4-hydroxy-tamoxifen/afimoxifene molecule toward *C*. *neoformans* (Fig 3, molecules 12–18) and, as a result, 4-fold more active than tamoxifen (molecule 1). This indicates that the hydrogen bonding properties of the hydroxyl substituent are not required for the improved activity of the 4-hydoxy group on the phenyl ring. Ester groups in this position of the ring system function as electron withdrawing substituents. Thus, our data suggest that increasing the electronegative properties of the molecules improve anti-cryptococcal activity. Consistent with that hypothesis, a tamoxifen derivative with an electronegative fluorine substituent on the B ring is 4-fold more active than tamoxifen (Fig 3, molecule 19). Therefore, it appears that electronegative moieties on either ring A or B improves the antifungal activity of tamoxifen.

The anti-cryptococcal activity of the acylated 4-hydroxy-tamoxifen derivatives was consistent for acetyl, phenyl, and substituted phenyl esters (Fig 3, molecules 12–15). Further increasing the electronegative properties of the ester with bromo- and cyano-substituted phenyl esters (Fig 3, molecules 16 & 17) did not improve activity relative to the sterically similar 4-methyl-substituted analog (Fig 3, molecule 15). Finally, ester groups of various sizes are tolerated at this position; however, the very large biphenyl ester (Fig 3, molecule 18) is inactive, indicating that there are some steric constraints on substituents at the 4-position. Taken together, these data suggest that increasing the electronegative properties of the aromatic substituents of triphenylethylene derivatives leads to a modest, but consistent increase in their anti-cryptococcal activity with variable effects on their anti-candidal activity.

### The size of the substituent at the 2-position of the tamoxifen double bond plays an important role in antifungal activity

**Fig 4:**
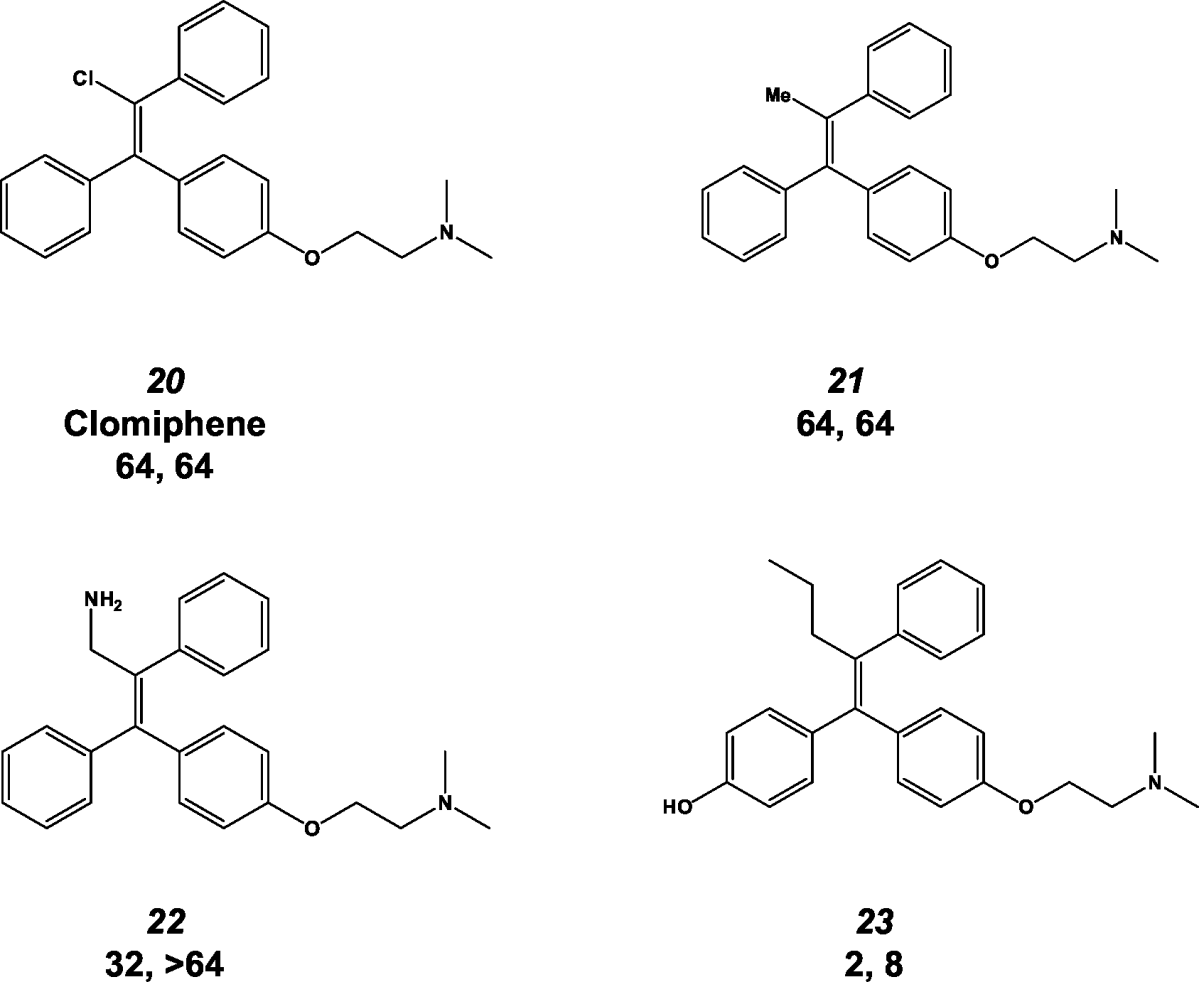
Antifungal activity of 2-ethenyl-substituted tamoxifen derivatives. The structure, molecule number used in the text, minimum inhibitory concentration (MIC, μg/mL) against *C*.*neoformans* (first number) and MIC against *C*. *albicans* are provided.

The final position of the tamoxifen scaffold that we probed with our set of molecules was the aliphatic substituent of the double bond (Fig 1). Tamoxifen has an ethyl group at this position while toremifene has a chloroethyl moiety; the activity of these two derivatives is identical (Fig 2, molecules 1 & 4). All of the commercially available SERMs with antifungal activity also have an alkyl substituent at this position. Clomiphene (Fig 4, molecule 20), a commercially available SERM that has a chloride instead of an alkyl substituent at this position, is poorly active against both *C*. *neoformans* and *C*. *albicans*. Similarly, a tamoxifen derivative with a methyl instead of an ethyl group at the double bond position was essentially inactive against *C*. *neoformans* and *C*. *albicans* (Fig 4, molecule 21). This suggests that the size/length of the substituent at the double bond plays an important role in the antifungal activity of the scaffold. Further supporting this hypothesis is the fact that a derivative of 4-hydroxy-tamoxifen with a propyl side chain is 2-fold more active (Fig 4, molecule 23) than the ethyl-containing 4-hydroxy-tamoxifen (Fig 2, entry 2). The derivative containing a methylamino substituent on the double bond (Fig 4, molecule 22) is slightly more active than the corresponding methyl derivative but not as active as a molecule with an ethyl substituent, indicating that aliphatic substituents are more effective than charged amino substituents at this position. Although we have a small number of molecules in this series, it seems clear that the size of the substituent at the 2-position of the double bond plays an important role in antifungal activity of the tamoxifen scaffold.

### The anti-cryptococcal activity of tamoxifen derivatives correlates with ability to interfere with calmodulin dependent Crz1 nuclear localization

**Fig 5:**
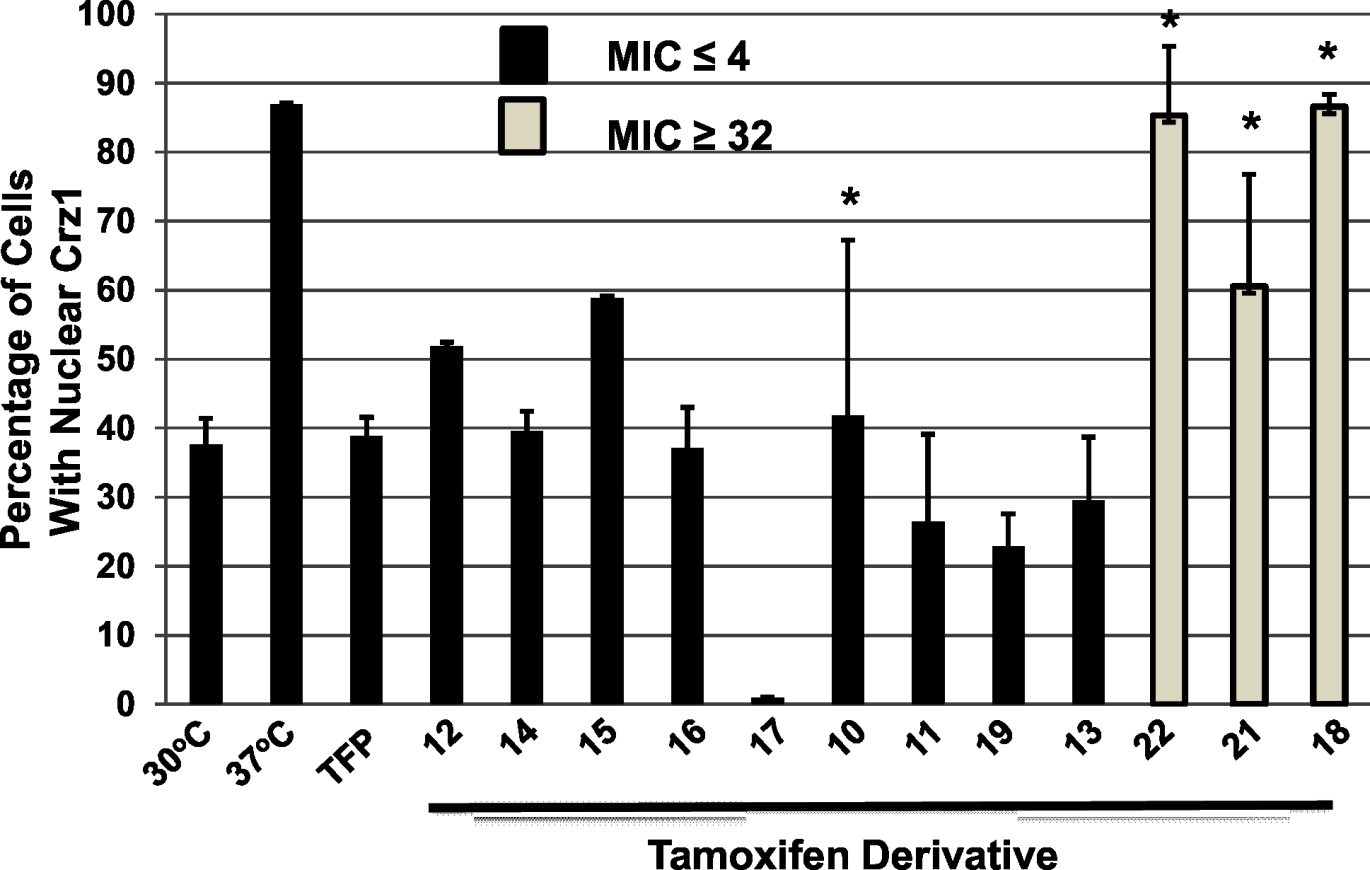
Effect of tamoxifen-derivatives on the temperature-induced, calmodulin-dependent nuclear localization of the transcription factor Crz1. As described in the materials and methods, *C*. *neoformans* cells harboring Crz1-mCherry and the nuclear marker Nop1-GFP were shifted to 37°C in the presence or absence of the indicated tamoxifen derivative (see Figs 2–4 for structures corresponding to the molecule numbers) at ¼ of minimum inhibitory concentration (MIC). The percentage of cells with co-localized mCherry and GFP signals was determined for a minimum of 100 cells with at least two independent biological samples. The bars indicate mean percentage of cells with nuclear Crz1 and error bars indicate standard deviation. All compounds except those with an asterisk (*) above the bar gave values that were significantly different than the untreated control (P< 0.05, Student’s unpaired, two-tailed t test). MIC values are taken from data in Figs 2–4.

Tamoxifen and its derivatives are well-characterized inhibitors of calmodulin function [21] and we have shown that calmodulin inhibition is an important contributor to the mechanism of the antifungal activity of triphenylethylenes [12, 18]. Based on these results, we hypothesized that tamoxifen derivatives with good antifungal activity (MIC 2–8 μg/mL) would inhibit calmodulin function while those with poor activity (MIC 32–64 μg/mL) would not. To test this hypothesis, we used a cell-based reporter assay developed previously in our laboratory that allows us to monitor the localization of the transcription factor Crz1p [12]. Under conditions of heat stress in *C*. *neoformans*, calmodulin binds to, and activates, the protein phosphatase calcineurin [22]. Calcineurin, in turn, dephosphorylates Crz1p which triggers its translocation to the nucleus were it is responsible for stress related gene expression [23]. The assay is based on a *C*. *neoformans* reporter strain that expresses both Crz1p-mCherry and Nop1p-GFP, a nucleolar protein, as a nuclear marker. When *C*. *neoformans* is incubated at 30°C, Crz1p is predominately localized in the cytoplasm with 38% of cells showing nuclear localization (Fig 5). Upon shifting cultures to 37°C, the proportion of cells with nuclear Crz1p increases to 87% (Fig 5). We have previously shown that this process is blocked by calmodulin antagonists [12] including the well-characterized calmodulin inhibitor trifluopromazine (Fig 5).

As shown in Fig 5, tamoxifen derivatives with MIC >32 μg/mL were poor inhibitors of heat stress induced-Crz1p nuclear localization. For these molecules [18, 21, 22], the percentage of cells with nuclear Crz1p in the treated samples did not differ significantly from the untreated samples (P> 0.05, Student’s unpaired, two-tailed t test). In contrast, all molecules except for 10 (Fig 2) with MIC < 4 μg/mL induced a statistically significant decrease in the percentage of cells with nuclear Crz1 (Fig 5). The main outlier for this trend was molecule 10 (Fig 2); this is due to the fact that the data were quite variable compared to other examples. We have a relatively small set of molecules with MIC values that segregate into two groups: good or poor activity. Therefore, it is not possible to generate a detailed analysis of the relationship between molecular structure and on-target activity without molecules displaying a broader range of activities. Ideally, we would have had enough of each compound to generate EC_50_ curves but that was not the case. However, our observations are consistent with the notion that tamoxifen analogs that inhibit calmodulin-dependent process in *C*. *neoformans* cells also have good anti-cryptococcal activity.

As we have described previously [12, 18] and as supported by our data in Fig 5, the antifungal activity of the triphenylethylene class is due in part to their ability to interfere with calmodulin. The ability of tamoxifen to interfere with mammalian calmodulin activity is well-established and may contribute to the estrogen receptor independent effects of this class of molecules [21]. Based on these observations, molecular modeling studies have been used to gain further insights into the structure-activity relationships between the triphenylethylene structure and mammalian calmodulin antagonism [24]. In order to better understand how the antifungal activity of the tamoxifen scaffold correlated with calmodulin antagonism, we generated a homology model of *Cryptococcus neoformans* var. *grubii* calmodulin (*CAM1*, CNAG_01557) based on the structure of bovine calmodulin co-crystallized trifluoperazine, a well-characterized calmodulin antagonist [25]. The sequences in the region near the ligand binding sites of the bovine and *C*. *neoformans* Cam1 are nearly identical, differing by only one residue; therefore, the model is expected to be reliable.

**Fig 6:**
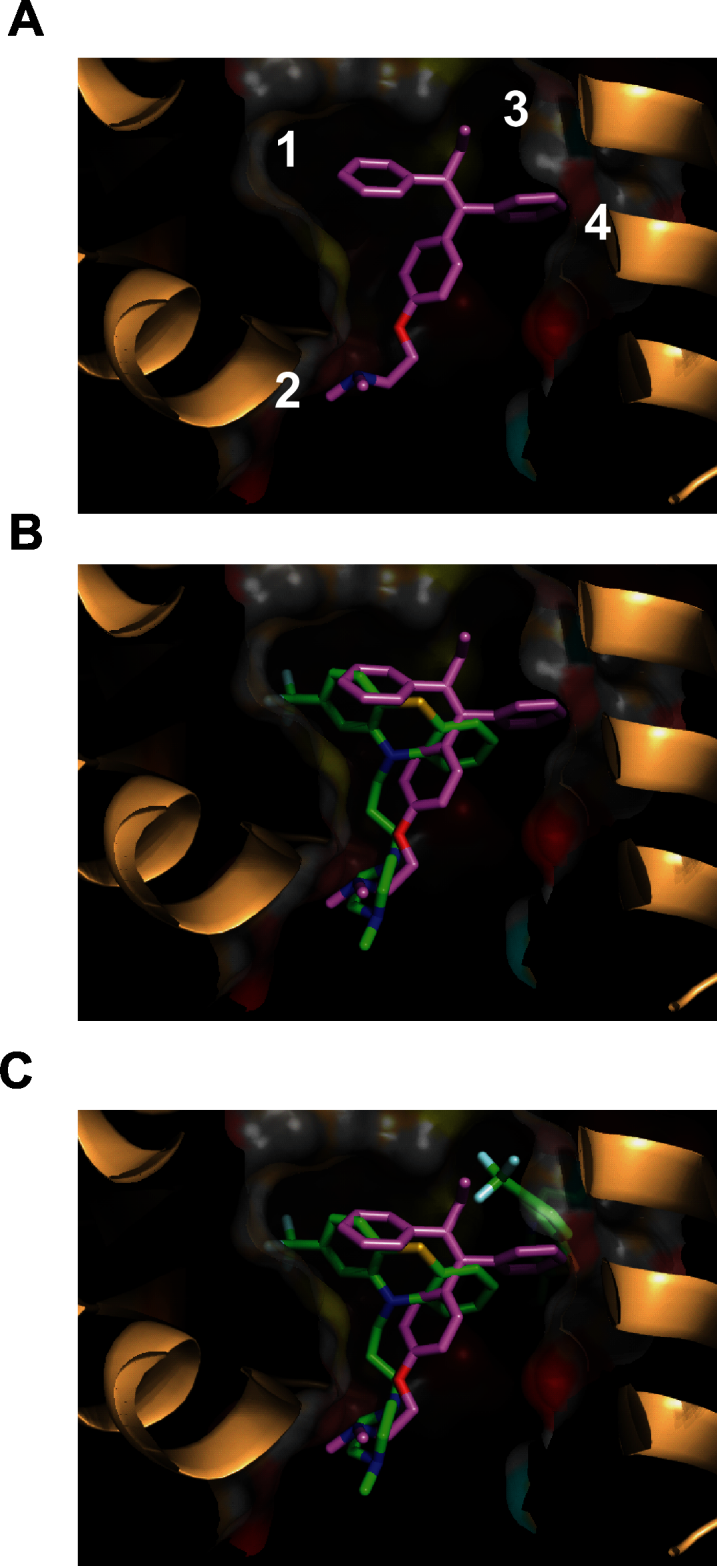
Model of tamoxifen binding to *Cryptococcus neoformans* calmodulin. As described in the materials and methods, a homology model of *C*. *neoformans* calmodulin bound to tamoxifen was built based on the structure of the bovine calmodulin-trifluoperazine complex [25]. A. Binding of tamoxifen to the trifluoperazine binding pocket. The numbers are arbitrary indicators of the four regions of the binding pockets described in the text. B. Overlay of tamoxifen with one molecule of trifluoperazine in the binding pocket. C. Overlay of tamoxifen with binding pocket containing both molecules of trifluoperazine.

Tamoxifen was modeled in the *Cn*Cam1 binding pocket (Fig 6A) defined as the first two sites occupied during titration of bovine calmodulin with trifluoperazine [25]. The four regions of the tamoxifen molecule interact with four regions of the trifluoperazine-binding pocket as indicated in Fig 6A. The resulting binding models indicate that the B and C rings of tamoxifen occupy the same regions of the binding pocket as the aromatic core of the first trifluoperazine molecule in the bovine calmodulin structure (Fig 6B). Consistent with the trifluoperazine-structure [25], the alkoxy-amino substituent on the aromatic C ring of tamoxifen is positioned analogously to the alkyl-piperazine substituent of trifluoperazine (Fig 6C). As noted by Cook et al. [26], the tethered alkyl-piperazine substituent of trifluoperazine is oriented toward the solvent-exposed region of the binding pocket and appears to interact with a cluster of negatively charged glutamate residues on helices I and IV. Our model indicates that the alkoxy-dimethylamino moiety of tamoxifen interacts with *Cn*Cam1 in the same manner (Fig 6A, region 2). Modeling studies of the interaction of tamoxfien with mammalian calmodulin also suggested that the alkylamino group interacts with acidic residues [24]. Consistent with this model, ospemiphene (Fig 2, molecule 9) an analog of toremifene in which the basic alkylamino group has been replaced by a hydroxyl group, is devoid of antifungal activity. Thus, our antifungal activity data and modeling indicate that the requirement for an alkylamino group in this series of compounds is related to interactions with the acidic residues near the binding pocket.

In summary, molecular modeling of the interactions of tamoxifen derivative with a homology model of *Cryptococcus* calmodulin provides a structural rationale for the observed activities of these molecules and allows the formation of hypotheses regarding the subsequent optimization of the scaffold’s anti-cryptococcal activity.

## Discussion

The antifungal activity of SERMs related to tamoxifen has been known for a number of years [17]. Our laboratory has shown that tamoxifen combines with fluconazole to reduce fungal brain burden in a mouse model of cryptococcosis and is active in a mouse model of disseminated candidiasis [12, 18]. Although SREMs are active against both *Cryptococcus* and *Candida* spp., the data reported here indicate that the activity of the scaffold against *C*. *albicans* is somewhat erratic. Across closely related molecules, the activity of the scaffold against *C*. *neoformans* varies only slightly (see Fig 2) while the activity against *C*. *albicans* varies substantially with seemingly minor changes. One explanation for this observation is that the activity of the tamoxifen derivatives against *C*. *albicans* may vary with factors such as cellular penetration or uptake rather than specific molecular interactions. If that is the case, then the relationships between antifungal activity and structure may not be readily apparent from inspection of the structure of the molecules. As noted in the introduction and summarized below, the tamoxifen scaffold is much more attractive as a potential treatment for cryptococcosis than for other fungal infections. As such, we will focus the remainder of the discussion on the anti-cryptoccocal activity of the tamoxifen-related scaffold.

**Fig 7:**
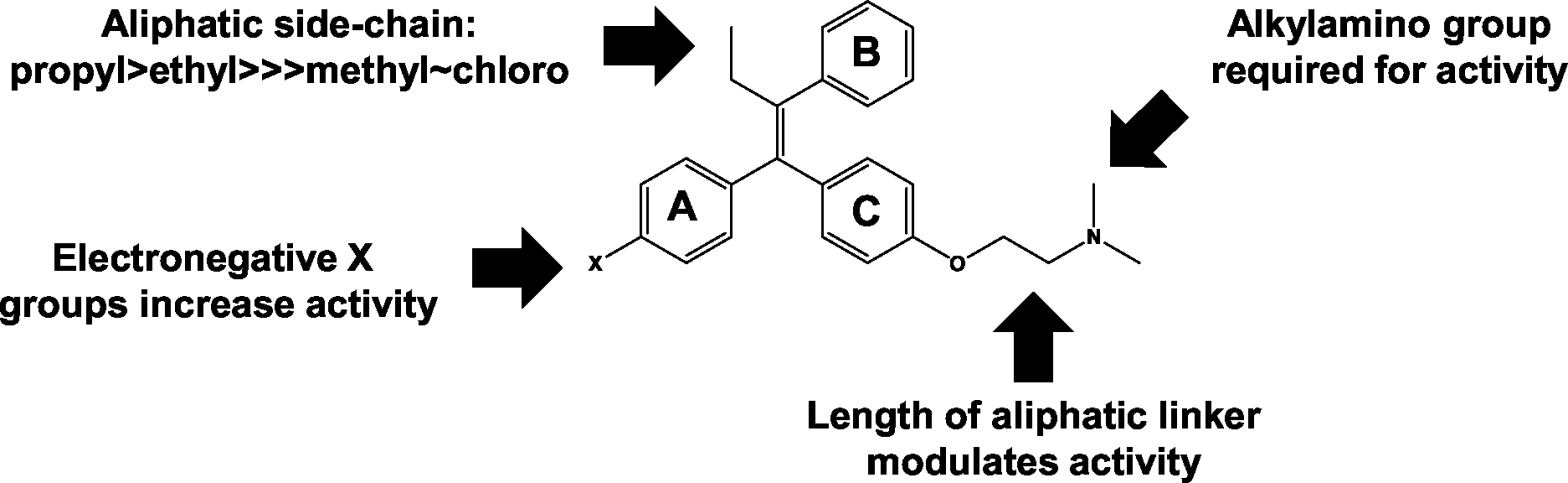
Summary of structure-activity relationships.

From our set of commercially available SERMs and tamoxifen derivatives from the AstraZeneca archive (Fig 3), we have identified four important features of the relationship between the structure of triphenylethylene-based SERMs and anti-cryptococcal activity; these are summarized in Fig 7. First, aliphatic substituents longer than one carbon at the 2-position of the double bond improve activity substantially. Second, the presence of a basic group such as an amine tethered to the one of the aromatic rings is required for activity. Ospemiphene (Fig 2, molecule 9) is a potent estrogen receptor antagonist but has no antifungal activity and, thus, it appears that the structural requirements for these two activities are distinct. Third, our data indicate that electronegative substituents on the aromatic rings increase the anti-cryptococcal activity relative to analogs without such functionality. Fourth, previous studies by our group have shown that extending the length of the aliphatic linker between the C ring phenoxy group and the alkylamino moiety improves anti-cryptococcal activity [12]. Although the set of analogs is not extensive, these data provide the first insights into which specific features of the tamoxifen scaffold are important for antifungal activity and, thereby, represent regions where further optimization can be focused.

We previously reported that extending the linker in the context of the tamoxifen analog idoxifen (Fig 2, molecule 5) improved the antifungal activity [12]. Hardcastle et al. found that increasing the length of the alkyl group linking the amino group to ring C of tamoxifen improved the calmodulin antagonism in vitro [24]. Our modeling results suggest that the increasing the length of the tether may improve interactions between the positively charged amine moiety and the negatively charged acidic residues near the binding pocket (Fig 6A, region 2). Hardcastle et al. [24] found that an 8 carbon linker was optimum for calmodulin inhibition. Thus, their data and our modeling suggest that further modifications to the length of the tether may lead to additional improvements in the anti-cryptococcal activity of the tamoxifen scaffold.

Our data and modeling also suggest a second region of the tamoxifen scaffold that may be amenable to further optimization. The binding model indicates that the aliphatic side chain of tamoxifen fits into a narrow, hydrophobic groove in the binding site (Fig 6A, region 3). The anti-cryptococcal activity of the triphenylethylene scaffold is exquisitely sensitive to alterations at this position (Fig 4). A chloro (Fig 4, molecule 20) or methyl (Fig 4, molecule 21) group at this position appears to be too small or does not extend far enough into the pocket to establish a strong interaction. Further supporting this notion is the fact that the methyl-substituted analog (Fig 4, molecule 21) is a poor antagonist of calmodulin-mediated Crz1 nuclear localization (Fig 5). The activity of molecules with ethyl groups (e.g., tamoxifen, molecule 1) and chloro-ethyl (e.g., toremifene, molecule 4) indicate that these substituents are tolerated. Comparison of the propyl-substituted 4-hydroxyl-tamoxifen derivative (Fig 4 molecule 23) to 4-hydroxy-tamoxifen (Fig 2, molecule 2) indicates that extension of the side-chain from ethyl to propyl improves activity modestly (2-fold). Interestingly, the positively charged amino-ethyl substituent in molecule 22 (Fig 4, molecule 23) is much poorer than similarly sized neutral substituents with respect to both anti-cryptococcal activity and ability to block Crz1 nuclear localization. A reasonable explanation for this observation is that charged groups such as the amino moiety of molecule 22 do not interact as well with the hydrophobic pocket as neutral substituents. It may be possible to further optimize the interactions between substituents at this position and the binding pocket.

Finally, our data indicate that a wide variety of sterically diverse substituents at the 4-position of the aromatic A ring of the tamoxifen scaffold are well tolerated. For example, an analog containing the small 4-acetoxy substituent (Fig 3, molecule 12) differs little in activity from an analog with the much bulkier 2-ethyl benzoyloxy group (Fig 3 molecule 14); only the extremely bulky biphenyl ester 18 shows reduced activity (Fig 3). The flexibility of this position appears to be due to the fact that this region of the scaffold occupies the secondary TFP binding site (Fig 6A, region 4) and, consequently, allows the larger groups to extend into the solvent [25]. Hardcastle et al. found that electronegative substituents such as 4-iodo groups improve calmodulin antagonism [24]. Our data indicate that electron withdrawing 4-acyloxy substituents on the A ring modestly improve antifungal activity (Fig 3) and ability to block Crz1 nuclear localization; however, the structural basis for this improvement is not evident from our models.

It is important to point out that, while the calmodulin model provides a useful framework upon which to analyze the structure-activity data and base the design of new triphenylethylene analogs, there are significant limitations to the conclusions that can be drawn. For example, we have shown that improving calmodulin antagonism improves antifungal activity. It is, however, important to consider that calmodulin is unlikely to be the only cellular target contributing to the antifungal activity of the triphenylethylenes. Indeed, we have found that triphenylethylenes interact with Cml1p, an EF-hand containing protein in *C*. *neoformans* with homology to both calmodulin and centrin-family proteins [12]. Additional EF-hand-containing proteins are present in the *C*. *neoformans* genome and, although we have not observed binding to other members of this class, it remains possible that interaction of tamoxifen-like molecules with multiple EF-hand containing targets contributes to their antifungal activity [12]. Furthermore, the triphenylethylene scaffold is known to have additional “off-target” activities that could also contribute to the structure-activity relationships observed for the scaffolds anti-cryptococcal activity [19]. Nevertheless, these data and analyses indicate that improving the calmodulin antagonism of the tamoxifen scaffold improves antifungal activity and, consequently, provide a structure-based framework for the further optimization of the scaffold as a potential antifungal drug.

## Materials and Methods

### Strains and media

*C*. *neoformans* var. *grubii*, strain H99 stud (Serotype A, Matα) and *C*. *albicans* SC5314 were used for all in vitro susceptibility experiments. Crz1 localization experiments were performed with XW252 (H99, MATα, *CRZ1*-mCherry::*NEO GFP-NOP1*::*NAT*, [12]). The strains were cultivated on yeast peptone dextrose (YPD) plates and used within two weeks. *C*. *neoformans* var. *grubii* was grown in YPD medium containing 2% (wt/vol) dextrose, 2% (wt/vol) Bacto-peptone and 1% (wt/vol) yeast extract. YPD agar plates contained 2% (wt/vol) Bacto-agar at 30°C or 37°C.

### Estrogen antagonists and tamoxifen analogs

Commercially available drugs and molecules were obtained from Sigma (St. Louis, MO) and used as received. The tamoxifen analogs from the Astra Zenca molecular archive were generously provided by Dr. Alita Miller (Astra-Zeneca, Cambridge, MA). All molecules have been previously reported in the scientific or patent literature and were >95% pure by LC-MS. Molecules were dissolved in 100% DMSO (10 mg/mL) and stock solutions were stored at -20°C until use.

### In vitro antifungal susceptibility testing

*C*. *neoformans* var. *grubii* and *C*. *albicans* susceptibility testing was performed accordance with Clinical and Laboratory Standards Institute (CLSI, [27]) protocols using RPMI 1640 medium (with glutamine and phenol red, without bicarbonate, Invitrogen) buffered to pH 7.0 with 0.165 M 3-(*N*-morphilino)-propanesulfonic acid. All wells contained 200 μL of media, 1000 cells, and 2-fold serial dilutions of the drug along one plate axis (range from 0–64 μg/mL). DMSO was used as the solvent for all drugs (final DMSO concentration 0.1%). Plates were incubated at 37°C for 48–72 hr and the minimum inhibitory concentration (MIC) was defined as the lowest drug concentration in which no growth was visible using criteria described by CLSI [27]. Individual susceptibility assays were carried out in triplicate for each drug concentration on at least two separate occasions.

### Crz1 localization

The effect of small molecules on the temperature-induced nuclear localization of Crz1 was determined as previously described [12]. Briefly, overnight cultures of strain XW253 containing Crz1p-mCherry and Nop1p-GFP in YPD at 30°C were diluted 1:50 into fresh YPD and mock treated (1% DMSO) or treated with ¼ the MIC of the test compound. The resulting cultures were incubated for an additional 4 hr at either 30°C or 37°C. Samples (1 mL) were harvested by centrifugation, washed with DPBS, re-suspended in DPBS, and imaged using a Nikon ES80 epi-fluorescence microscope equipped with a CoolSnap CCD camera. Images were collected using NIS-Elements Software and processed in PhotoShop. Each condition was imaged on multiple days from independent cultures. For quantitative analysis, at least 100 cells with clearly visible signals for both flurophores were analyzed for each replicate and the percentage of cells with colocalized Crz1p-mCherry and Nop1p-GFP were calculated. Data are presented as mean percentage with error bars representing standard deviation.

### *C*. *neoformans* calmodulin homology and ligand binding models

The sequence for *C*. *neoformans var*. *grubii* calmodulin (*CAM1*) was downloaded from the Broad *C*. *neoformans* var. *grubii* H99 database (ORF CNAG_01557; http://www.broadinstitute.org/annotation/genome/cryptococcus_neoformans/MultiHome.html) and was aligned to a structure of bovine calmodulin bound to two molecules of the calmodulin antagonist trifluoperazine (Protein Database Structure 1A29; http://www.rcsb.org/pdb/explore.do?structureId=1a29; [25]). A homology model was built using the AMBER12/EHT force field and standard protocols with MOE 2013.08 from CCG. The starting ligand structures were imported as structure data files (SDF) into MOE 2013.08 using Wash. Parameterization and low mode 3D conformer search (Molecular operating environment (MOE), 2013.08; Chemical Computing Group Inc., Montreal, Canada). For each ligand the lowest energy 3D conformer was iteratively aligned to the two trifluoperazine ligands of structure 1A29 using the MOE process for rigid and flexible alignment. Initial poses were evaluated for consistency with the antifungal structure-activity relationships. Best poses were optimized in the homology binding site with MOE LigX to generate the final binding models.
